# Effects of Fluticasone Propionate on *Klebsiella pneumoniae* and Gram-Negative Bacteria Associated with Chronic Airway Disease

**DOI:** 10.1128/msphere.00377-22

**Published:** 2022-11-07

**Authors:** Lesa A. Begley, Kristopher Opron, Guowu Bian, Ariangela J. Kozik, Cai Liu, Jeremy Felton, Bo Wen, Duxin Sun, Yvonne J. Huang

**Affiliations:** a Division of Pulmonary and Critical Care Medicine, Department of Internal Medicine, University of Michigan, Ann Arbor, Michigan, USA; b Pharmacokinetics and Mass Spectrometry Core, College of Pharmacy, University of Michigan, Ann Arbor, Michigan, USA; c Department of Microbiology and Immunology, University of Michigan, Ann Arbor, Michigan, USA; University of Texas Medical Branch

**Keywords:** inhaled corticosteroids, bacteria, microbiome, asthma, COPD, corticosteroids

## Abstract

Inhaled corticosteroids (ICS) are commonly prescribed first-line treatments for asthma and chronic obstructive pulmonary disease (COPD). Recent evidence has shown that ICS use is associated with changes in the airway microbiome, which may impact clinical outcomes such as potential increased risk for pneumonia in COPD. Although the immunomodulatory effects of corticosteroids are well appreciated, whether ICS could directly influence the behavior of respiratory tract bacteria has been unknown. In this pilot study we explored the effects of fluticasone proprionate, a commonly prescribed inhaled corticosteroid, on respiratory bacteria with an expanded focus on Klebsiella pneumoniae, a species previously implicated in fluticasone-associated pneumonia in COPD. We observed significant effects of fluticasone proprionate on growth responses of *K. pneumoniae*, as well as other bacterial species isolated from asthmatic patients. Fluticasone-exposed *K. pneumoniae* displayed altered expression of several bacterial genes and reduced the metabolic activity of bronchial epithelial cells and their expression of human β-defensin 2. Targeted assays identified a fluticasone metabolite from fluticasone-exposed *K. pneumoniae* cells, suggesting this species may be capable of metabolizing fluticasone proprionate. Collectively, these observations support the hypothesis that specific members of the airway microbiota possess the functional repertoire to respond to or potentially utilize corticosteroids in their microenvironment. These findings lay a foundation for novel research directions into the potential direct effects of ICS, often prescribed long term to patients, on the broader airway microbial community and on the behavior of specific microbial species implicated in asthma and COPD outcomes.

**IMPORTANCE** Inhaled corticosteroids are widely prescribed for many respiratory diseases, including asthma and COPD. While they benefit many patients, corticosteroids can also have negative effects. Some patients do not improve with treatment and even experience adverse side effects. Recent studies have shown that inhaled corticosteroids can change the make-up of bacteria in the human respiratory tract. However, whether these medications can directly impact the behavior of such bacteria has been unknown. Here, we explored the effects of fluticasone propionate, a commonly prescribed inhaled corticosteroid, on Klebsiella pneumoniae and other airway bacteria of interest, including primary species isolated from adult asthma patients. We provide evidence of growth responses to direct fluticasone exposure in culture and further examined fluticasone’s effects on K. pneumoniae, including gene expression changes and effects of fluticasone-exposed bacteria on airway cells. These findings indicate that members of the human airway bacterial community possess the functional ability to respond to corticosteroids, which may have implications for the heterogeneity of treatment response observed clinically.

## INTRODUCTION

Asthma and chronic obstructive pulmonary disease (COPD) are two highly prevalent airway diseases characterized by chronic inflammation and episodic symptoms of shortness of breath and cough, typically treated with inhaled medications that include corticosteroids (ICS) (1). It is well known that corticosteroids modulate host gene expression and immune responses to dampen inflammation (1). While the use of ICS is an effective treatment for many patients, clinical response to ICS is variable, even among patients with clinical biomarkers or phenotypes expected to benefit from ICS. Prolonged ICS exposure, particularly at higher doses, can also have adverse side effects (2). In COPD patients, ICS use, in particular fluticasone-containing preparations, has been associated with increased risk of pneumonia (3–6).

Host pathways modulated by corticosteroids have been extensively studied (1). Relative immunosuppression by systemic corticosteroids can elevate infection risk, but this is less certain with inhaled preparations and could be dose related. Even in the absence of a clear acute infection, growing evidence from respiratory microbiome studies indicates that ICS treatment is associated with changes in the lower airway microbiome of asthma and COPD patients (7–10). In a randomized, placebo-controlled clinical trial of inhaled fluticasone propionate administered to subjects with mild asthma for 6 weeks (7, 8), we found that fluticasone treatment altered lower airway microbial composition, particularly in nonresponders whose airway physiology did not improve with fluticasone. We further observed that the baseline airway microbiome of fluticasone nonresponders was enriched in predicted bacterial functions that included pathways for xenobiotic and drug metabolism (7).

These clinical observations led us to further explore whether corticosteroids used as inhaled treatments for asthma or COPD might directly affect the functional behavior of airway bacteria, a largely unstudied topic. Supportive evidence that bacteria could directly respond to or transform steroidal compounds come from environmental microbiology studies (11), where the capacity of environmental bacteria to metabolize sex steroids (12, 13) and the presence of steroid catabolism genes in a wide range of environmental microbial metagenomes have been described (14). We focused first on Gram-negative airway bacteria (e.g., members of the *Proteobacteria* phylum) that not only can cause opportunistic pneumonia, but also become more prevalent in the airway microbiome of patients with more severe asthma or COPD where ICS treatment is common (15–18). Interestingly, studies of bacteria with a steroid-processing functional repertoire have largely been based on analyses of environmental sources of *Proteobacteria* and *Actinobacteria*, two phylogenetically distinct phyla that we previously observed in the bronchial microbiome of severe asthma subjects to be associated with contrasting clinical outcomes and airway epithelial gene expression profiles (19).

Here, we report findings from a pilot study to determine whether a clinically relevant, Gram-negative respiratory bacterium can respond to ICS and to characterize functional consequences of this exposure. We focused on Klebsiella pneumoniae, a species that has been implicated in ICS-associated pneumonia in clinical trials of fluticasone-based therapies for COPD and whose clearance can be impaired by presence of fluticasone (20, 21). An earlier *in silico* analysis of *K. pneumoniae* genomes also has suggested the presence of corticosteroid catabolism genes in this species (22). We conducted culture-based experiments wherein fluticasone propionate was provided as the only predominant carbon source available. We evaluated *K. pneumoniae* growth and transcriptional responses, effects of fluticasone-conditioned *K. pneumoniae* on airway epithelial cells and explored the fate of fluticasone in *K. pneumoniae* cultures using target metabolite assays. Lastly, we further explored whether other Gram-negative respiratory bacteria, including primary strains isolated from sputum of asthmatic patients, are able to grow in fluticasone-enriched culture.

## RESULTS

### Growth response to fluticasone propionate of three representative respiratory pathogens.

We first examined growth responses of ATCC strains representing three opportunistic respiratory pathogens (Klebsiella pneumoniae ATCC 13883, Pseudomonas aeruginosa ATCC 27853, and Stenotrophomonas maltophilia ATCC 13637). Quantification was performed by plating and colony counting at time points specific to each isolate to allow for different growth rates across species. Compared to a vehicle-only control, all three species demonstrated significant growth in the presence of 50 μM fluticasone proprionate: K. pneumoniae by 175% (*P* = 0.027; Student *t* test), P. aeruginosa by 244% (*P* = 0.008), and S. maltophilia by 263% (*P* = 0.032). Because of the clinical relevance of K. pneumoniae in the setting of ICS treatment as mentioned earlier, we pursued further experiments using *K. pneumoniae* ATCC 13883 to investigate fluticasone effects on *K. pneumoniae* behavior.

### Capacity of *K. pneumoniae* to metabolize fluticasone proprionate.

We first interrogated the publicly available genome sequence data for ATCC 13883 for presence of enzymes involved in steroid catabolism. Using pBLAST to search for each enzyme of the KEGG steroid biodegradation pathway (KEGG pathway 00984) within the ATCC 13883 protein complement, we identified multiple proteins involved in the degradation of steroid, such as testosterone, to the final breakdown product, HIP-CoA (Table 1). This information, complemented by findings from an earlier *in silico* analysis of *K. pneumoniae* genomes (22), provided biological plausibility for the hypothesis that *K. pneumoniae* ATCC 13883 may possess the functional repertoire to metabolize corticosteroids like fluticasone.

**TABLE 1:** Enzymes involved in steroid catabolism identified in the K. pneumoniae ATCC 13883 strain genome

Enzyme	EC no.	Primary UniProt query	ATCC 13883 NCBI accession no.
3β-Hydroxy-Δ5-steroid dehydrogenase	1.1.1.145	P9WQP6	KHF67732.1
3-Oxosteroid 1-dehydrogenase	1.3.99.4	Q8RLT5	KHF65792.1
3-Ketosteroid 9α-monooxygenase	1.14.15.30	A0A117NVS4	KHF52267.1
3-Hydroxy-9,10-secoandrosta-1,3,5(10)-triene-9,17-dione monooxygenase	1.14.14.12	P9WJA1	KHF52034.1
3-Alkylcatechol 2,3-dioxygenase	1.13.11.25	P9WNW7	KHF64954.1
3-[(3aS,4S,7aS)-7a-methyl-1,5-dioxo-octahydro-1*H*-inden-4-yl]propanoate–CoA ligase	6.2.1.41	P96843	KHF66761
3-Oxo-5α-steroid 4-dehydrogenase	1.3.99.5	JC6030	KHF65792.1
3(or 17)β-Hydroxysteroid dehydrogenase	1.1.1.51	A0A024HKT7	KHF64101.1

To test whether *K. pneumoniae* could metabolize fluticasone proprionate, we used both bacterial cells and supernatants generated from overnight cultures of *K. pneumoniae* cultured in M9 minimal medium with either fluticasone proprionate or vehicle only (DMSO). Based on available knowledge on fluticasone propionate metabolism in mammalian systems, we assayed for the parent compound and its two known metabolites: fluticasone 17β-carboxylic acid proprionate (M1) and fluticasone 17β-carboxylic acid (M2) (23). There was detectable accumulation of fluticasone 17β-carboxylic acid proprionate in the cellular fraction of *K. pneumoniae* that was exposed to fluticasone in culture (Table 2). The other known metabolite was not detected, and none of the assayed compounds were detected in samples from the vehicle-only condition. Similar results were obtained when Tween 80 was used as the vehicle solvent for fluticasone proprionate (data not shown).

**TABLE 2:** Measurement of fluticasone propionate and its known metabolites from K. pneumoniae and extracellular compartments

Condition and compartment	Mean level ± SEM^*a*^		
	Fluticasone proprionate (FTP)	Fluticasone 17β-carboxylic acid (M2)	Fluticasone 17β-carboxylic acid proprionate (M1)
Vehicle			
Cell pellet	Undetected	Undetected	Undetected
Supernatant	Undetected	Undetected	Undetected
Fluticasone			
Cell pellet	254,333 ± 43,143 ng/g	Undetected	20.87 ± 3.09 ng/g
Supernatant	33.6 ± 4.95 ng/mL	Undetected	Undetected

a Data represent the means of triplicate biological experiments.

### Fluticasone proprionate exposure alters gene expression in *K. pneumoniae*.

To explore how fluticasone propionate might impact *K. pneumoniae* gene expression, we performed bacterial RNA sequencing (RNA-seq) using *K. pneumoniae* cells harvested after 6 h of growth in fluticasone-supplemented minimal medium or vehicle-only conditions. Of the 5,125 transcripts obtained, 1,474 did not align with known genes. Eighty-three identified transcripts differed between fluticasone-exposed and nonexposed *K. pneumoniae*: 58 genes displayed higher expression, and 25 genes displayed lower expression in fluticasone-exposed *K. pneumoniae* (Table 3; *P* < 0.05). We observed the following trends in this exploratory analysis. Among the top genes with increased expression were *eptB* (log_2_ fold change [Log2FC] 1.53), a gene involved in antibiotic resistance mechanisms through modification of lipid A in lipopolysaccharide, *pstS* (log2FC 1.36; phosphate-binding protein in ABC transporter complex), *birA* (log2FC 1.28; DNA-binding transcriptional repressor/biotin-[acetyl-CoA-carboxylase] ligase), and *rpsB* (Log2FC 1.14; rRNA protein). Additional *K. pneumoniae* transcripts that increased with fluticasone exposure included genes for an inner membrane transport protein (ydhP_2; Log2FC 1.14), plasmid replication (*repB*), oxidative stress mitigation (*ahpC*, *grxD*, and *sodB*), and the regulation/suppression of biofilm (*bssS*). *K. pneumoniae* genes that showed decreased expression with fluticasone included rRNA-related genes (*rpmE2* and *rlmM*; Log2FC −3.27 and −1.66), an alpha-galactosidase (*galA;* Log2FC −1.60), and *bscB* (Log2FC −0.67) that is part of the cellulose/exopolysaccharide secretion system in biofilms (24). Multiple genes related to iron uptake and sequestration, including siderophore production (*entB*, *entA*, *entS*, *fiu*, and *hemH*), also showed decreased expression with fluticasone exposure (25).

**TABLE 3:** Differentially expressed genes in K. pneumoniae ATCC 13883 with fluticasone treatment^*a*^

Expression type and gene	Expression with fluticasone	
	Log2FC	*P*
Higher expression		
*eptB*	1.533	0.018
*pstS*	1.364	0.046
*birA*	1.284	0.027
*rpsB*	1.142	0.006
*ydhP_2*	1.136	0.035
*yjdJ*	1.086	0.007
*nusA*	0.974	0.030
*glyS*	0.958	0.045
*ftnA_2*	0.881	0.015
*osmB*	0.759	0.006
*cspE*	0.736	0.013
*yjhP*	0.726	0.025
*purL*	0.711	0.004
*ftnA_1*	0.657	0.020
*rpsJ*	0.650	0.023
*csrA*	0.643	0.000
*ylaC*	0.621	0.031
*raiA*	0.612	0.001
*dinJ*	0.603	0.030
*sodB*	0.588	0.043
*bssS*	0.558	0.039
*ybiV*	0.528	0.046
*dps*	0.451	0.000
*yobA_2*	0.439	0.028
*gntK*	0.429	0.042
*ymiA*	0.426	0.001
*ompX*	0.424	0.023
*uspG_2*	0.421	0.014
*ompC*	0.407	0.003
*rplM*	0.403	0.025
*speE*	0.398	0.012
*bglA_2*	0.371	0.033
*frr*	0.358	0.032
*ppiB*	0.355	0.037
*hldD*	0.347	0.017
*yqjC*	0.341	0.003
*hns_2*	0.338	0.023
*ynfD*	0.331	0.003
*spy*	0.330	0.009
*fnr*	0.320	0.043
*gapA*	0.303	0.003
*ompA*	0.302	0.005
*rplJ*	0.290	0.044
*ppa_2*	0.282	0.035
*lldR_2*	0.282	0.047
*arcA*	0.259	0.008
*nlpI*	0.254	0.020
*tolC*	0.251	0.029
*cyoA*	0.250	0.044
*yccA*	0.249	0.045
*grxD*	0.243	0.034
*ahpC_2*	0.242	0.034
*uspA*	0.241	0.044
*repB*	0.238	0.044
*folE*	0.232	0.030
*eno*	0.224	0.043
*rpoH*	0.223	0.041
*crp*	0.191	0.049
Lower expression		
*rpmE2*	–3.267	0.029
*rlmM*	–1.664	0.037
*thlA_2*	–1.599	0.046
*galA*	–1.597	0.021
*zinT*	–1.539	0.049
*fiu*	–1.337	0.026
*entB*	–1.180	0.021
*entA*	–1.048	0.034
*mtgA*	–0.940	0.009
*entS*	–0.931	0.018
*hscB*	–0.925	0.028
*wecB*	–0.893	0.045
*sutR_2*	–0.772	0.022
*bcsB_1*	–0.670	0.027
*cbiE*	–0.629	0.007
*fieF_2*	–0.603	0.018
*dipZ*	–0.537	0.049
*abgB*	–0.525	0.028
*mdtD_2*	–0.514	0.045
*hemH*	–0.489	0.037
*murG*	–0.360	0.040
*dxs_3*	–0.333	0.048
*lsrG*	–0.320	0.031
*chaC_2*	–0.295	0.018
*gloC*	–0.290	0.045

a RNA-seq was performed on biological triplicates of fluticasone-exposed versus vehicle-only exposed bacteria after 6 h of culture. The log_2_ fold change (Log2FC) between groups was determined using DESeq2. Transcripts reaching a threshold of *P* < 0.05 (unadjusted) from this exploratory analysis are shown.

**FIG 1:**
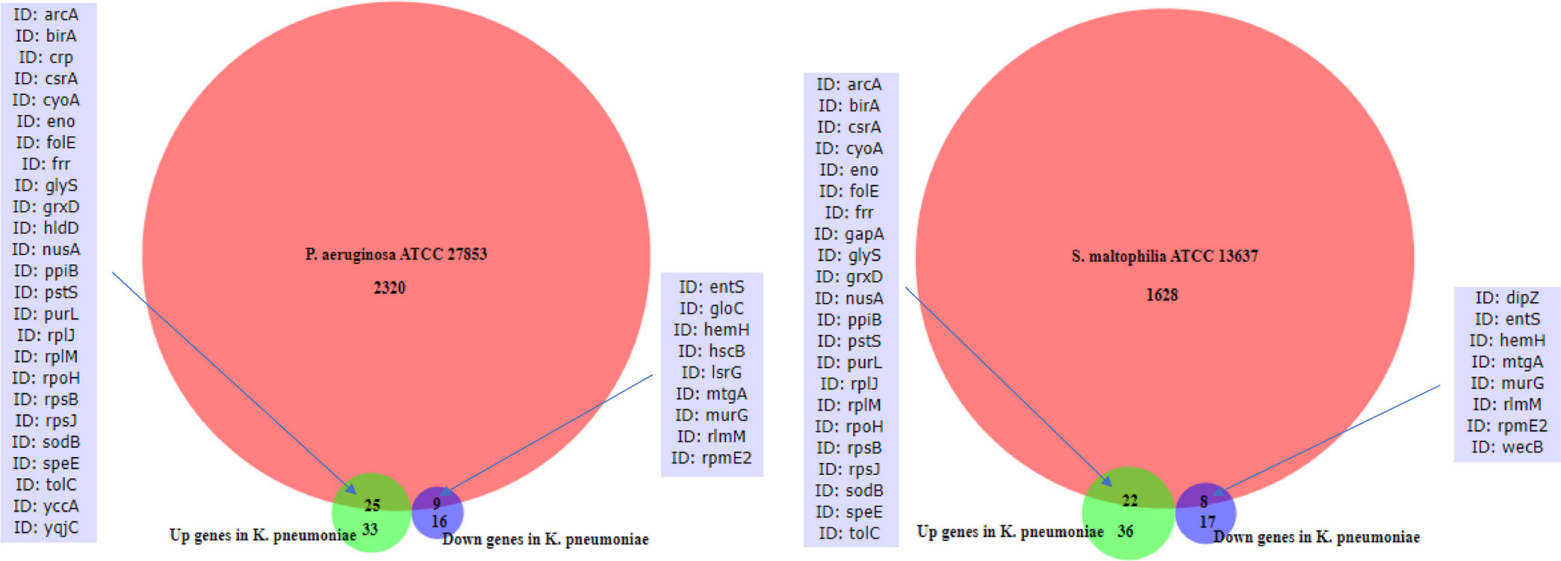
Presence in P. aeruginosa and S. maltophilia of same genes that were differentially expressed in fluticasone-treated K. pneumoniae.

We additionally examined whether the other two ATCC strains that demonstrated growth response to fluticasone propionate (P. aeruginosa and S. maltophilia) possess the same genes that were differentially expressed in *K. pneumoniae* in response to fluticasone. Overlap was observed based on analysis of their reference genomes (Fig. 1). P. aeruginosa ATCC 27853 possessed 25 and 9 of the genes that were up- and downregulated, respectively, in fluticasone-treated *K. pneumoniae*. S. maltophilia ATCC 13637 possessed 22 and 8 of the genes that were up- and downregulated, respectively, in *K. pneumoniae*. A majority of these overlapping genes were present in all three species. This suggests the possibility that functions conferred by these genes may be impacted by fluticasone across species.

### Fluticasone-conditioned *K. pneumoniae* alters airway epithelial cell activity and β-defensin gene expression.

**FIG 2:**
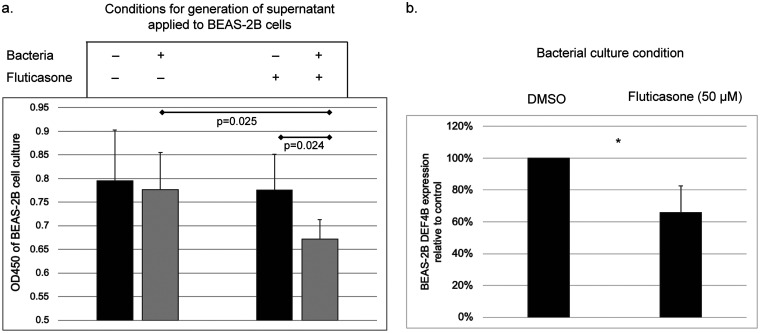
Effect of fluticasone-treated K. pneumoniae on bronchial epithelial cells. (a) Exposure of BEAS-2B cells to supernatants from fluticasone-treated *K. pneumoniae* culture, untreated *K. pneumoniae* culture, or control culture conditions. Reduced metabolic activity (WST-8 assay) was observed in cells exposed to supernatants from fluticasone-conditioned *K. pneumoniae*. Data represent results from triplicate experiments. (b) Epithelial cells exposed to fluticasone-treated, heat-inactivated *K. pneumoniae* displayed lower expression of β-defensin 2 (*DEF4B*), relative to vehicle control (*, *P* < 0.05 [*t* test]).

The airway epithelium acts as both a physical barrier and a source of innate immune effectors (26). To explore whether steroid-conditioned *K. pneumoniae* might affect epithelial cells differently from non-steroid-exposed *K. pneumoniae*, we utilized the human bronchial epithelial cell line BEAS2-B. Since bacterial effects on epithelial cells may occur via signals released extracellularly, we exposed BEAS2-B cells to supernatants from *K. pneumoniae* cultures under the same conditions as in the prior experiments (minimal media plus fluticasone or vehicle only). Control experimental conditions included exposing the epithelial cells to supernatants from uninoculated culture media supplemented with fluticasone or vehicle only or from inoculated culture media without fluticasone. Epithelial cells exposed to supernatant from fluticasone-conditioned *K. pneumoniae* culture displayed a significant decrease in cellular activity (WST-8 viability assay) compared to the other experimental conditions (Fig. 2a). These observations suggest that exposure of *K. pneumoniae* to fluticasone generates an altered extracellular environment that can repress metabolic activity of airway epithelial cells and possibly affect their viability.

Airway epithelial cells produce and release small anti-microbial peptides, such as defensins, that effect microbial killing. In the lung the predominant defensin is β-defensin 2 (*DEF4B*). We examined the expression of β-defensin 2 mRNA by BEAS-2B cells when exposed to *K. pneumoniae* that had been grown in minimal media with fluticasone prior to inactivation by heat killing. To control for any residual steroid presence, cells were also exposed to uninoculated medium with or without steroid. Expression of *DEF4B* was undetectable when cells were exposed to uninoculated media irrespective of steroid presence. Expression of *DEF4B* was detected when cells were exposed to heat-killed *K. pneumoniae*, but transcript levels from cells exposed to fluticasone-exposed, heat-killed *K. pneumoniae* were only 65.7% of the levels detected from epithelial cells exposed to *K. pneumoniae* cultured in vehicle alone (*P* = 0.01, Fig. 2b). This suggests that *K. pneumoniae* exposure to fluticasone dampens its effect in eliciting β-defensin 2 gene expression from airway epithelial cells.

### Fluticasone supports growth of other airway bacteria cultured from asthmatic subjects.

Recent studies have established associations between the composition of lower airway microbiota and clinical and inflammatory features in adult asthma (27). Having observed the responses of *K. pneumoniae* to fluticasone proprionate, we further explored the potential impacts of this corticosteroid on other members of the respiratory microbiota. Using primary strains of Gram-negative bacteria isolated from induced sputum of asthmatic patients, we examined their growth responses cultured under the same minimal medium conditions that were used to test *K. pneumoniae*, including vehicle controls. Species from multiple genera demonstrated statistically increased growth when exposed to fluticasone in culture (Table 4).

**TABLE 4:** Growth responses of primary strains of Gram-negative respiratory bacteria to fluticasone proprionate^*a*^

Organism	% growth change	*P*
*Achromobacter* spp.	19.0	0.004
*Acinetobacter baumannii*	347.6	<0.001
*Citrobacter koseri*	7.9	0.45
*Enterobacter* spp.	–2.0	0.013
*Escherichia/Shigella*	27.2	0.019
*Klebsiella* spp.	30.5	<0.001
*Klebsiella aerogenes*	12.9	0.001
*Klebsiella grimontii*	16.2	0.002
*Klebsiella oxytoca*	1.9	0.68
*Klebsiella variicola*	13.8	<0.001
*Pseudomonas* spp. (not *P. aeruginosa*)	–2.5	0.065
*Pseudomonas aeruginosa*	47.7	<0.001
*Serratia marcescens*	47.8	<0.001
*Stenotrophomonas maltophilia*	38.8	<0.001

a Experiments were repeated up to four times, and differences in growth were averaged over the experiments. Values were measured at the time of maximal response for each species. The statistical difference between vehicle and fluticasone conditions was determined using a Student *t* test.

## DISCUSSION

ICS are widely prescribed treatments for asthma or COPD but are not effective in every patient and can have important adverse effects. It has been unknown whether ICS could directly affect bacterial functional behaviors, despite recent studies showing that ICS treatment can alter the composition of airway microbiota (7–9). This would further implicate altered functional phenotype of airway microbes as an additional effect of ICS therapy, which could have negative or positive clinical consequences. To begin to address this question, we focused first on Gram-negative species, such as K. pneumoniae, which have a potentially pathogenic role in lung diseases. Among the Gram-negative isolates cultured from patients with mild to moderate asthma on ICS therapy, multiple strains displayed growth responses to fluticasone under minimal medium culture conditions compared to vehicle control. All of the tested species are members of the *Proteobacteria* phylum, which has been linked to more severe airway disease (27). Using K. pneumoniae to conduct more in-depth experiments, we observed additional effects of fluticasone on this species. Exposing *K. pneumoniae* to fluticasone resulted in altered expression of several bacterial genes and dampened the ability of *K. pneumoniae* to stimulate β-defensin 2 gene expression from human bronchial epithelial cells. Interestingly, we identified a known metabolite of fluticasone propionate from *K. pneumoniae* cells cultured in fluticasone-supplemented media, which was not found in the supernatants or in control test conditions. These novel observations collectively support possible off-target effects of fluticasone on the functional behavior of airway microbes. More broadly, these findings have additional, potentially important implications for the entirety of the airway microbiome, which shifts compositionally in more severe airway disease coinciding with increased prevalence of ICS use clinically.

Many studies have described the effects of ICS on host responses in the context of asthma or COPD (1, 10), but to our knowledge none have specifically explored direct effects of ICS on airway bacteria implicated in these diseases. While we used Klebsiella pneumoniae as a representative species to further interrogate steroid-induced effects, we also observed fluticasone effects on growth responses of several other Gram-negative species, albeit to various degrees. ATCC strains of Pseudomonas aeruginosa and Stenotrophomonas maltophilia also displayed greater growth response to fluticasone than most of the other primary bacterial strains tested from asthmatic patients. The experimental culture conditions used were designed to detect direct effects of fluticasone as the main carbon substrate present and thus were artificially restrictive. Nonetheless, the observations support the hypothesis that some airway species can respond directly to ICS in the absence of any host cell presence.

Growth response is only one readout of bacterial behavior, since changes in other types of functional responses may occur. To further explore this on a species-specific level to start, we dedicated further experimental efforts using K. pneumoniae. In contrast to prior murine studies describing fluticasone-induced suppression of airway cell host responses contributory to impaired pathogen clearance in acute infection models (21, 28), we sought instead to characterize the direct effects of fluticasone on *K. pneumoniae* using a strain that demonstrated significant growth response to this corticosteroid. Using available reference genome data, we identified coding sequences for multiple enzymes in the steroid degradation pathway and in support of this functional repertoire, identified a known fluticasone metabolite from *K. pneumoniae* cells cultured in fluticasone-supplemented media. This suggests that this particular *K. pneumoniae* strain was able to uptake fluticasone and enzymatically alter the parent compound in the absence of any host cells. To our knowledge, this is the first evidence that a clinically important species may be able to utilize fluticasone as a substrate. Although the specific steps involved in this *K. pneumoniae* strain are unknown at this time, recent studies have catalogued the presence of xenobiotic and steroid metabolism genes across a variety of microbes found in the environment, including Klebsiella sp. and many other bacterial genera relevant to chronic airway diseases (14, 29).

Our exploration of *K. pneumoniae* transcriptional responses to fluticasone using RNA-seq identified several genes that were differentially expressed in fluticasone-exposed *K. pneumoniae* compared to non-fluticasone-exposed *K. pneumoniae*. The results suggest altered functional phenotype of *K. pneumoniae* when exposed to fluticasone. For example, the *galA* gene, which is involved in capsule biosynthesis, demonstrated reduced expression upon fluticasone exposure. The biofilm suppressor gene *bssS* was upregulated, while a gene involved in biofilm production was downregulated. Multiple genes involved in scavenging iron were also downregulated. Upregulation of bacterial genes involved in plasmid replication and mitigation of oxidative stress was also observed which could help with bacterial survival or growth, as was observed for this particular strain with fluticasone. Altogether, these patterns suggest fluticasone-associated changes in bacterial fitness and a less virulent phenotype, which could result in reduced provocation of innate immune responses in the airway and support of bacterial survival.

Results from experiments using airway epithelial cells suggest that fluticasone-conditioned *K. pneumoniae* may elicit different epithelial cell responses compared to non-fluticasone-exposed *K. pneumoniae*. In addition to decreased metabolic activity, the epithelial cells also displayed lower expression of β-defensin 2, an important human airway antimicrobial peptide, when exposed to fluticasone-conditioned *K. pneumoniae*. Reduced airway levels of antimicrobial peptides have been reported in COPD patients with more advanced disease and been associated with concurrent ICS use (28, 30, 31). Our experimental results here implicate fluticasone-induced changes in bacterial functional phenotype as a possible indirect effect of corticosteroids on host cell responses.

Our overall intent in this pilot study was to establish initial evidence that airway bacteria can respond directly to corticosteroids in their milieu. While these results lay a foundation for further research, we acknowledge several limitations. The minimal media culture conditions utilized were artificially restrictive to support our primary intent and clearly do not mimic the richer and more complex airway environment. We also focused on screening growth responses of Gram-negative airway bacteria to fluticasone and used a specific strain of *K. pneumoniae* for more detailed experiments. Further studies that expand upon this would be of interest, including determining corticosteroid effects on other airway microbiota from multiple phyla. Recent evidence from clinical studies implicates non-*Proteobacteria* involvement in milder COPD or asthma (7, 8, 32). Cumulative use of ICS over time might have important consequences on other microbiota members as well. While ICS use in COPD presumably suppresses inflammatory immune responses that drive acute exacerbations, direct effects of ICS on airway microbes could counteract this by fostering bacterial survival and other functional traits that benefit specific species in the lung. The present study also reports findings from our testing of just one corticosteroid compound, but other types of inhaled corticosteroids also are commonly prescribed. Lastly, future studies using more complex host models, such as mice, would be of interest to determine whether and how steroid-conditioned bacteria modify host immune responses *in vivo*.

In summary, we show that members of the airway bacterial microbiota, including species with known pathogenic potential, display functional capacity to respond directly to fluticasone propionate, a commonly prescribed inhaled corticosteroid. Although the clinical consequences of ICS-induced alterations in airway microbiome functions are not yet known, further research could uncover microbe-specific mechanisms induced by corticosteroids that potentially play a role in mechanisms of airway disease progression.

## MATERIALS AND METHODS

### Culture isolation of primary respiratory bacteria.

Bacteria were isolated from induced sputum collected from asthmatic subjects as part of a prospective observational study approved by the University of Michigan Institutional Review Board (Characterization of Adults for Asthma Microbiome Research Studies; NCT02887911). All subjects provided written, informed consent. Raw sputum was plated onto sheep blood and MacConkey agars. Gram-negative isolates from MacConkey agar were identified using 16S rRNA (rRNA) gene sequencing, coupled with motility and urease assays when necessary. After pure cultures were isolated, bacteria were lysed by boiling for 15 min, and the resulting lysate was used as the PCR template, along with the 16S rRNA universal primer pair 27F/1492R. Amplicons were submitted for Sanger sequencing, and the resulting sequences were analyzed using BLAST (https://blast.ncbi.nlm.nih.gov/Blast.cgi) against the 16S rRNA reference library. Species calls of 97% or higher identity in BLAST were considered definitive. If multiple species reached this threshold for a given sequence the organism was reported at the genus level.

### Bacterial growth assays.

For testing of primary bacterial strains, fresh overnight cultures grown aerobically in Luria broth (LB; Becton Dickinson) were inoculated into minimal (M9) medium plus 0.05% glucose with either 50 μM fluticasone proprionate or equivolume solvent (Tween 80; Sigma-Aldrich; final concentration, 0.25%) and then plated in sextuplicate in round-bottom 96-well plates. Plates were incubated at 37°C with shaking, and growth was assessed by determining the optical density at 600 nm hourly for up to 12 h; uninoculated media were plated as controls. For growth assays testing ATCC strains (Klebsiella pneumoniae ATCC 13883, Pseudomonas aeruginosa ATCC 27853, and Stenotrophomonas maltophilia ATCC 13637), overnight aerobic cultures in LB (37°C at 150 rpm) were inoculated into M9 medium plus 0.05% glucose with 50 μM fluticasone proprionate or equivolume solvent (DMSO; final concentration, 0.25%; Sigma-Aldrich). Serial dilutions were plated onto LB agar in triplicate at 4, 8, 12, and 24 h to yield 100 to 200 colonies per plate and counted the following day.

### *K. pneumoniae* RNA-seq.

A fresh overnight culture of *K. pneumoniae* (ATCC 13883) was used to inoculate M9 minimal medium supplemented with 0.05% glucose and either 50 μM fluticasone proprionate or equivolume DMSO. Each condition was grown in triplicate flasks for 6 h at 37°C with shaking and cell pellets collected. Cell pellets were resuspended in lysis buffer and RNA extracted using an RNeasy minikit (Qiagen). The resulting RNA was treated with DNase I (Thermo Fisher) per the manufacturer’s instructions to remove any remaining DNA and purified by overnight ethanol precipitation. RNA was prepared for sequencing by the Advanced Genomics Core at the University of Michigan, which included steps to deplete rRNA prior to the preparation and sequencing of 50-bp libraries on the Illumina platform. The raw sequences were assessed and processed with FastQC and Trimmomatic. Reads were mapped to the *K. pneumoniae* transcriptome and counted using Hisat2 (33) and HTseq-count (34). Differential analysis was performed with DEseq2 (35).

### Evaluation for potential metabolism of fluticasone propionate by *K. pneumoniae*.

A fresh overnight culture of *K. pneumoniae* (ATCC 13883) was used to inoculate 100 mL of M9 medium plus 0.05% glucose and 50 μM fluticasone proprionate or equivolume solvent (DMSO; final concentration, 0.25%). After 16 h of growth, the cultures were centrifuged, and the supernatant was collected and centrifuged a second time. Then, 10 μL of centrifuged supernatant was plated onto LB agar to test for the complete removal of bacterial cells, and the remaining volume was frozen at −80°C. Cell pellets were weighed and frozen at −80°C until being assayed for fluticasone propionate and metabolites.

Measurement of fluticasone propionate (FTP), fluticasone 17β-carboxylic acid propionate (M1), and fluticasone 17β-carboxylic acid (M2) concentrations in the cell pellet and supernatant fractions was conducted by the University of Michigan College of Pharmacy Pharmacokinetics and Mass Spectrometry Core. FTP, M1, and M2 levels were determined by a liquid chromatography-tandem mass spectrometry (LC-MS/MS) method developed and validated for this study. The LC-MS/MS method consisted of a Shimadzu HPLC system, and chromatographic separation of tested compound was achieved using a Waters XBridge-C_18_ column (5 cm × 2.1 mm, 3.5 μm). The mobile phases were 0.1% formic acid in purified water (A) and 0.1% formic acid in acetonitrile (B). The gradient (B) was held at 10% (0 to 0.5 min), increased to 99% at 1.0 min, remained at isocratic 99% B for 2.0 min, and was then immediately stepped back down to 10% for a 2-min reequilibration. The flow rate was set at 0.4 mL/min. Under these conditions, all compounds eluted at ~1.8 min. An AB Sciex QTrap 5500 mass spectrometer equipped with an electrospray ionization source (Applied Biosystems, Toronto, Canada) in the positive-ion multiple reaction monitoring (MRM) mode was used for detection. FTP, M1, M2, and the internal standard fluorometholone were detected using the MRM transitions 501.0→293.1 *m/z*, 453.0→293.1 *m/z*, 397.0→357.1 *m/z*, and 377.0→279.1 *m/z*, respectively. All mass spectrometry parameters for ion detection were optimized automatically on instrument. Optimized parameters enable quantitation of the FTP concentrations over the linear range of 25 to 10,000 ng/mL and the M1 or M2 concentration over the linear range of 1 to 1,000 ng/mL with weighted linear regression (1/×2). The linearity of the relationship between the peak area ratio and the concentration was demonstrated by the correlation coefficients (*r* > 0.995). Data were collected and integrated with Analyst software, version 1.62. FTP, M1, and M2 were quantified by comparison of their peak areas (determined by mass spectral analysis) to those of analytical standards.

### Airway epithelial cell activity assay and measurement of β-defensin 2 expression.

Supernatants from *K. pneumoniae* cultures grown under the same conditions described earlier (M9 medium plus 50 μM fluticasone or equivolume DMSO; 37°C for 16 h) were passed through a 0.22-μm-pore-size filter, and 10 μL of filtered supernatant plated on LB agar to verify complete removal of bacterial cells. Uninoculated culture media were incubated and processed alongside.

The human airway epithelial cell line BEAS-2B (ATCC CRL-9609) was maintained in bronchial epithelial growth medium (BEGM; Lonza) on collagen-coated tissue culture plastic. Cells were seeded into 96-well plates ~16 h prior to exposure to supernatants from either fluticasone-conditioned *K. pneumoniae* culture or a culture medium control. Seeded epithelial cells were washed once with phosphate-buffered saline, followed by the addition of bacterial supernatant or control that was mixed with an equal volume of BEGM, with eight replicate wells per experimental condition. After 16 h of incubation at 37°C, the epithelial cell activity was assessed using the WST8 reagent according to the manufacturer’s instructions.

For measurement of β-defensin 2 expression, a fresh culture of *K. pneumoniae* was inoculated into M9 media plus 0.05% glucose with either 50 μM fluticasone proprionate or equivolume DMSO solvent (final concentration, 0.25%) and grown for ~16 h. Bacteria were heated at 70°C for 20 min to kill the cells but maintain cellular integrity. Heat killing was verified by plating 10 μL on LB agar, and cellular integrity was verified by microscopy. Heat-killed bacteria were added to BEAS-2B cultures at ~60% confluence in triplicate. After 8 h, the cells were washed and harvested in TRIzol reagent (Ambion), and RNA was extracted according to the manufacturer’s instructions. One microgram of total RNA was treated with DNase I prior to reverse transcription. Assays for β-defensin 2 (*DEF4B*, ABI Hs00823638_m1) and a control gene (*GAPDH*, ABI Hs99999905_m1) were used, along with TaqMan reagents, to measure gene expression, and the ΔΔ*C_T_* values were calculated.

### Data availability.

The RNA sequence data for K. pneumoniae (ATCC 13883), fluticasone treated versus untreated, have been deposited in the NCBI Sequence Read Archive under accession number PRJNA863279.
